# The Role of Creative Publicity in Different Periods of the COVID-19 Outbreak in China: Taking the Creative Publicity of Chinese Poetry as an Example

**DOI:** 10.3389/fpsyg.2021.600818

**Published:** 2021-02-12

**Authors:** Dandan Jia, Cuicui Sun, Zhijin Zhou, Qingbai Zhao, Quanlei Yu, Guanxiong Liu, Yi Wang

**Affiliations:** ^1^Key Laboratory of Adolescent Cyberpsychology and Behavior (Central China Normal University), Ministry of Education, Wuhan, China; ^2^School of Psychology, Central China Normal University, Wuhan, China

**Keywords:** COVID-19, Chinese poetry, creative publicity, general publicity, periods

## Abstract

When humans are confronted with an epidemic situation or a continuous natural disaster, success depends largely on how critical information is conveyed to as many people as possible, how individuals' emotional experiences of the crisis are elicited, and how their behaviors are directed going forward. Efficient publicity is key to successful epidemic prevention and control. This study explores the role of creative publicity by comparing the influence of creative publicity and general publicity in different periods of the COVID-19 outbreak in China. The effects of creative and general publicity differed across varying periods of the COVID-19 outbreak. Specifically, compared to general publicity, creative publicity had a significant impact on individuals' emotional arousal in the early period of the COVID-19 outbreak while it significantly promoted individuals' emotional arousal, behavioral regulation, and willingness to actively disseminate information in the middle period. In the stable period of the COVID-19 outbreak, creative publicity performed better than general publicity in regulating individuals' behavior. On the other hand, general publicity was more effective than creative publicity in regulating individuals' behavior and actively disseminating information about the epidemic in the early period of the COVID-19 outbreak. In conclusion, creative and general publicity had differing effects in different periods of the COVID-19 outbreak, which may relate to the characteristics of the publicity format and people's psychological conditions in different periods of the COVID-19 outbreak.

## Introduction

The coronavirus disease (COVID-19) epidemic caused by severe acute respiratory syndrome coronavirus 2 (SARS-CoV-2) was discovered in the city of Wuhan, China, in December 2019 and spread rapidly around the world (Zhang et al., 2020). To effectively curb the spread of COVID-19, China gradually implemented strict containment measures such as “Wuhan lockdown,” “home quarantine,” “wear a mask,” and “maintain social distancing.” At the same time, countries worldwide are also taking measures to prevent the spread of the COVID-19 pandemic and protect the health of their citizens. However, because of differences in culture and values across countries, the forms of publicity differ, which may result in varied public understanding, acceptance, and implementation of “home quarantine” policies and, thus, greatly affect epidemic prevention and eventual control. In China, the COVID-19 outbreak began in Wuhan and spread rapidly across the country. From the lockdown of Wuhan on January 23, 2020, to the reopening of Wuhan on April 8, it took just 76 days to progress from the COVID-19 outbreak to ultimately having it under control. China's success in fighting the virus is mainly attributed to the adoption of strict containment measures and movement restrictions to cut off virus transmission and the active cooperation of the public. Effective publicity plays a significant role in ensuring people clearly comprehend the real situation of the COVID-19 pandemic, accept expert preventative health advice, take the initiative to regulate their own behaviors, and strictly implement various containment measures.

In our study, publicity is defined as the transmission and reporting of information, policies, knowledge and other contents under the guidance of official or authoritative departments. Its main purpose is to give people access to knowledge and information as much as it is to raise awareness and regulate their own behavior (e.g., Ehrenberg et al., 2002; Yuningsih and Suherman, 2020). Publicity can take the form of slogans, media reports, etc. In this study, there are similarities and differences between publicity and advertising. The main difference is the ultimate purpose. The ultimate goal of advertising is to guide consumers to purchase behavior. The two publicity processes are very similar, mainly through the psychological impact of people to achieve the ultimate purpose of publicity. Therefore, our research will refer to relevant theories and results in the advertising field.

The motto of Stephan Vogel, Chief Creative Director of Ogilvy and Mather Germany, is “Nothing is more efficient than creative advertising. Creative advertising is more memorable, lasts longer, spends less on the media, and can be faster to build a fan group.” Creative publicity is highly valued for its ability to attract more attention and convey information in an entertaining or challenging way (Singam et al., 2014). What is most needed in the event of natural disasters, serious accidents, and crises is to disseminate information in a highly effective manner and to maintain free-flowing information to the public (Spence et al., 2015). Therefore, we believe that, in the case of natural disasters such as the COVID-19 pandemic, dissemination of information may achieve a multiplier effect with half the effort with the adoption of creative publicity forms. There have been numerous studies in the field of advertising in which the effects of publicity have been evaluated.

Heinz M Goldman, an international marketing expert, once proposed a classic AIDAS model to investigate advertising effects (Strong, 1925). The impact of advertising on people was mainly divided into five steps: Attention, Interest, Desire, Action and Satisfaction. He believed that creative publicity must attract people's attention or change to the publicity content. This would steer people to be interested in the advertised content; thus, generating customers' desires which would in turn create purchase behaviors to ultimately attain a deal. At present, it is still the most commonly used theoretical basis for studying the psychological effect of advertisements (Ansari and Joloudar, 2011; Shehata, 2016; Takaya and Yamashita, 2020). The quality of an advertisement can be tested by its advertising effect. Lavidge and Steiner (1961) six-stage theory of advertising effectiveness had a great impact on the advertising field. The theory proposed that advertising would influence people's awareness, knowledge, liking, preference, conviction, and purchases. The model subdivided cognition, emotion, and behavior into three stages. For example, cognition was divided into two subtle stages: awareness and knowledge, emotion into liking and preference, and behavior into conviction and purchase (Wang, 2008, p. 8). Creativity is the soul and heart of advertising (Tevi and Koslow, 2018). Research has found that creative advertising is more effective at motivating people to purchase products than simply listing product attributes or benefits. Creative information can attract more attention and lead to a positive attitude toward the products being sold (Reinartz and Saffert, 2013). Additionally, creative advertising is more popular and likable than traditional advertising (Demir, 2017). Theories about likeability suggest that, if the public likes an advertisement, they will pay more attention to the content of the advertisement, carry out in-depth processing of relevant information, and generate more positive perceptions of the brand at the same time (Stone et al., 2000). Relevant research also shows that likes and positive emotional experiences interact (Madden et al., 1988). Creative advertisements are most likely to induce people's positive emotional reactions (Bilby et al., 2020). At the same time, creative ads are more likely to attract people's cognitive processing, attention and recall effects than ordinary ads (Huhmann and Limbu, 2020). In short, creative publicity have better advertising effect than general publicity. creative advertising can not only affect the public's emotional experience (Aaker and Stayman, 1990) but also change the public's perception of the brand and, to a certain extent, affect their purchasing behavior (Ansari and Joloudar, 2011; Ahn et al., 2012; Sameti and Khalili, 2017).

At present, most research in the field of creative publicity examines creative advertising. Novelty is the cornerstone of creative advertising and that distinguished it from general advertising (Ang et al., 2014). Novelty refers to an advertisement containing novel, different and unusual elements (Smith and Yang, 2004). To embody novelty, some creative advertisements incorporate cultural elements, such as mythological structures (Johar et al., 2001). In addition, creative advertising should be meaningful and connected to the public so they can feel it is relevant and useful to them (Ang et al., 2007). Therefore, a successful creative advertisement mainly includes two characteristics: novelty and usefulness (Ang et al., 2007; Kilgour and Koslow, 2009; Arden et al., 2010; Runco and Jaeger, 2012).

Poetry, an outstanding cultural tradition in China, comprises concise and vivid language with poetic features (e.g., rhyme and meter) and is widely used in people's daily lives. Poetry as a creative language, from religious ceremonies and speeches to commercial advertisements, language with poetic stylistic features has long been preferred (Obermeier et al., 2016). Studies have shown that poetic style can promote individuals' cognitive fluency (Reber et al., 2004) and is easier to remember and disseminate. At the same time, poetic style itself can easily arouse people's emotions and empathy (Lüdtke et al., 2014). In short, the style of poetry is not only novelty and originality, but also funniness, so it is more popular. Poetic style applied to the publicity regarding natural disasters, serious accidents, or crisis events, such as the COVID-19 pandemic can be regarded as a kind of creative text publicity.

According to AIDAS model of advertising effect (Strong, 1925) and the six-stage theory (Lavidge and Steiner, 1961), advertising acts as a guide to influence the behavior of consumers or audiences by attracting people's attention, emotion, and cognition. Creative advertising is more likely to attract people's attention because of its novelty (e.g., Reinartz and Saffert, 2013; Ang et al., 2014). It is also more popular among people, evoking positive emotional experiences (e.g., Stone et al., 2000; Demir, 2017; Bilby et al., 2020). The poetic style itself has a certain novelty and interest (e.g., Reber et al., 2004), and has a good mass foundation, which is well-liked by people (Obermeier et al., 2016). Therefore, we assume that creative publicity based on a poetic style may impact citizens' emotion, cognition and behavior at different periods of the COVID-19 outbreak. From the early detection of SARS-CoV-2 to the period of severe outbreaks and then a certain degree of control, the challenges faced in each period differ, and how to finely divide such periods may vary from country to country. We adopted Fink's crisis model (1986), which has been cited frequently, to classify the different periods of the COVID-19 outbreak in China.

The key to the definition of a crisis is that crises can function simultaneously as threats and opportunities (Massey and Larsen, 2006). Fink (1986) described a crisis as “an unstable time or state of affairs in which a decision change is impeding” (p. 15) and proposed a method of dividing the lifecycle of a crisis into four stages. The first stage is the prodromal stage, in which indicators of the nature of the impending risk appear before the public and symptoms may appear. In the case of the COVID-19 outbreak in China, the indicator of the prodromal period is the “Wuhan lockdown” event, which represents the beginning of China's COVID-19 outbreak. Massey and Larsen (2006) pointed out that the pre-crisis stage provides opportunities for problem management; it can be assumed that, as the pre-crisis stage develops toward the crisis outbreak, the uncertainty-reduction process will further promote the demand for information. Therefore, at this stage of crisis development, emergency management personnel should increase publicity to make more people aware of possible crises. Currently, there is an urgent need for crisis information. After this stage, another trigger event marks the beginning of the second stage of the crisis, called the acute stage. In this stage, it is quite clear that a crisis is occurring, and vulnerable individuals or organizations suffer economic loss or damage to health. For the acute stage of COVID-19 in China, China has seen a sharp rise in daily confirmed cases. After the Chinese government implemented the “Wuhan lockdown” policy to slow the spread of the pandemic, which achieved remarkable results, other regions in China also followed the example of restricted “closures.” The Chinese economy “pressed the pause button” as the whole country began to implement the “home quarantine” policy, indicating the acute stage of COVID-19 (the middle stage) in China. Helsloot and Ruitenberg (2004) proposed that the public consciously taking effective measures in the acute stage of a crisis is the key to approaching challenges. In the middle period of the COVID-19 outbreak, the public is mainly fearful and anxious about the pandemic, and certain information (e.g., knowledge about medical data or self-protection) is urgently needed. To manage the pandemic, the Chinese government adopted policies such as “home quarantine,” “wear a mask,” “maintain social distancing,” and “health monitoring.” The effectiveness of epidemic prevention and control generally depends on the acceptance and implementation of these policies. Efficient promotion of prevention strategies and policies to the public is important. Therefore, a comprehensive publicity should be enacted at this stage to ensure people accept and implement relevant policies.

The third stage of crisis development is called the chronic stage. At this stage, the economies in some regions may have been damaged for a long time, and people must work hard to recover. As the number of COVID-19 cases in China gradually decreased, people from different regions began to return to work, and students went back to school. For China, the “Wuhan Unlocked” event on April 8, 2020, marks the onset of the third period of the COVID-19 outbreak, called the stable period. Fink (1986) theorized that a crisis ends in a termination stage during which it reaches a certain degree of resolution (see Mitroff, 1988). However, we consider that the current COVID-19 pandemic in China has not entered the final period of termination. Only after the Chinese people have been treated with an effective vaccine can China enter the period of termination. Therefore, our research focuses only on the first three periods of COVID-19 occurrence in China (early, middle, and stable).

In conclusion, the COVID-19 pandemic is a durative natural disaster event. In different periods, the effects of different forms of publicity may be differently affected by mental states, social environment, and informational needs. In previous studies, creative publicity was mainly used for product publicity. No empirical research on applying creative publicity to crisis events and exploring the effects of creative publicity has yet been found.

The study was to introduce an efficient medium to communicate with the public during different stages of crisis. This medium of communication will be loved and accepted by the public. Moreover, it can play a role in regulating people's behavior, and achieve the effect of quick and efficient communication with the public. By adopting this efficient communication method, public power can be quickly concentrated and the crisis can be dealt with swiftly. The COVID-19 pandemic is a crisis event for the public. Creative publicity was utilized as an effective means of communication to guide pandemic prevention and control. During the COVID-19 pandemic in China, we attempted to draw comparisons between the effects of general publicity and creative publicity forms across different periods of the COVID-19 pandemic, so as to further demonstrate the effects of the two forms in the publicity of crisis events and provide a strategy for efficient communication in such situations. Specifically, the study mainly focused on the following questions: (1) Is there any difference between the effect of general publicity and creative publicity in the three stages of the COVID-19 outbreak (early, middle, and stable periods)? (2) During the COVID-19 pandemic, will the adoption of creative publicity forms affect people's cognition, emotion, and behavior in a similar manner to creative advertising? (3) What factors will affect the impacts of the two forms of publicity?

**Figure 1:**
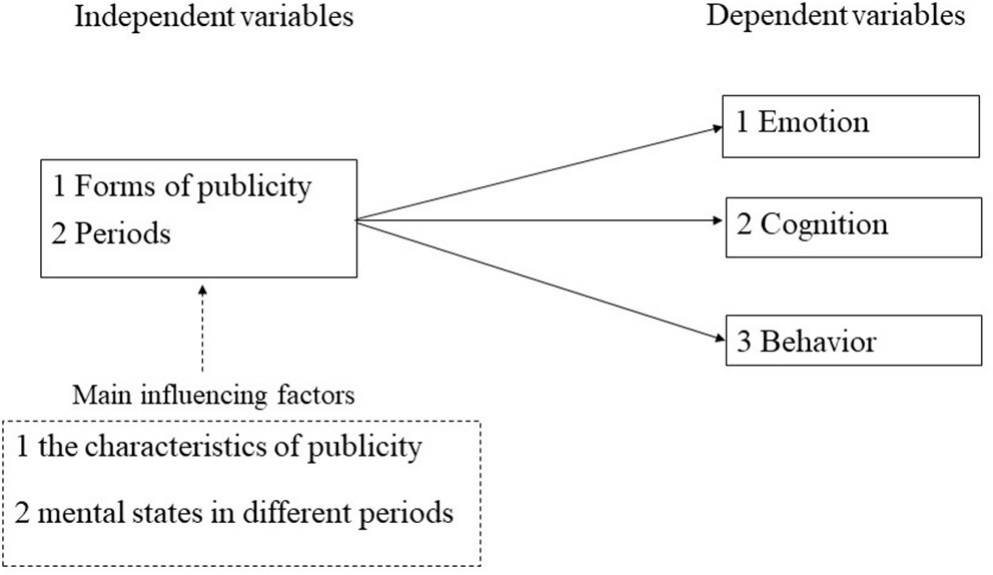
Experimental design and logical diagram of this study.

Depending on the psychological state across different periods of crisis (e.g., Perry, 1983; Helsloot and Ruitenberg, 2004) and the characteristics of creative publicity forms (e.g., Stone et al., 2000; Reinartz and Saffert, 2013; Ang et al., 2014; Demir, 2017; Bilby et al., 2020). We hypothesized that creative publicity of poetic style was significantly more effective than general publicity in influencing people's emotions, cognition and behavior during the early, middle, and stable period of the COVID-19 pandemic. See Figure 1 for the specific study design.

## Methods

### Material

After the outbreak of COVID-19 in China, we organized ten graduate students majoring in psychology from the creativity research team to collect the creative slogans from the Internet, print, social media and their daily lives about COVID-19. All members were trained in rating and selection of creative materials. We set standards for material selection: (1) Materials must be relevant to COVID-19. (2) The content selected is positive and in line with the national policy. (3) All materials are real in daily life. (4) Collect two forms of publicity with the same content but different forms (poetry style publicity; general publicity). Finally, the content, form and length of the two types of publicity were matched. We chose 40 pairs of materials for the initial experiment material.

We recruited participants online through an online questionnaire. Sixty participants who did not participate in the formal experiment were asked to rate the novelty and appropriateness of the two types of materials with seven points. According to the final novelty score, the top 30 percent of the experimental materials, 12 pairs were selected as the formal experimental materials. Statistical analysis of the final experimental materials was carried out and the results were found there was no significant difference in appropriateness, paired *t* (59) = −0.613, *p* = 0.524. A significant difference in novelty was found between creative (*M* = 5.46, *SD* = 0.68) and general publicity materials (*M* = 3.73, *SD* = 1.15), paired *t*_(59)_ = 10.95, *p* < 0.001, Cohen's *d* = 1.830. Poetic publicity was more novel as compared to general publicity. According to previous research, novelty is the core of creative publicity and that distinguished it from general publicity (Smith and Yang, 2004; Ang et al., 2007; Arden et al., 2010; Runco and Jaeger, 2012). We consider that the use of a poetic style of publicity is a kind of creative publicity. The material samples are listed in Table 1.

**Table 1:** Exemplar publicity: Take two kinds of publicity materials as an example.

**Publicity forms**	**Exemplar**
Creative publicity	武汉回来别乱跑, 传染病毒不得了 Wu han hui lai bie luan pao, chuan ran bing du bu de liao. Don't run around when you come back from Wuhan, it's very contagious.
General publicity	武汉回乡人员, 请主动隔离, 以防传染给其他人 Wu han hui xiang ren yuan, qing zhu dong ge li, yi fang chuan ran gei qi ta ren. Wuhan returnees, please take the initiative to quarantine, in order to prevent infection to others

*The experimental materials are given in Chinese pinyin and English*.

### Participants

We recruited participants online through random sampling and volunteer participation. A total of three hundred and thirty-four participants were obtained, all of whom were adults and native Chinese speakers. The participants were mainly students (75.10%) and employees (24.90%) from different regions of China except Wuhan.

The exclusion of Wuhan was based mainly on research considerations that in the early period of the epidemic in China. Wuhan was the epicenter of the early period of COVID-19 in China, and the pace of development of the epidemic may not be in line with that in other regions of China. In our research, the early period of the COVID-19 outbreak in China was based on the “Wuhan lockdown” as the starting point, so the development of the epidemic in Wuhan may have entered the early and middle periods of the epidemic earlier. Therefore, Wuhan was excluded from our study.

Finally, all participants gave informed consent and were rewarded after the experiment. Specific participant characteristics are shown in Table 2.

**Table 2:** The demographic characteristics of participants. *N* is the number of participants.

**Characteristics**	***N***	**Precent (%)**
Over all	334 (*M* = 28.59; *SD* = 7.88)	
Sex		
Male	134 (*M* = 29.07; *SD* = 7.89)	40.10
Female	200 (*M* = 28.27; S*D* = 7.88)	59.90
Age group		
18–30 years	252 (*M* = 24.60; *SD* = 3.61)	75.40
31–40 years	69 (*M* = 38.83; *SD* = 2.26)	20.70
41–50 years	11 (*M* = 49.73; *SD* = 0.47)	3.30
>50 years	2 (*M* = 52; *SD* = 0.00)	0.60
Education background		
High school graduate	16 (*M* = 35.11; *SD* = 8.33)	4.80
College graduate	266 (*M* = 25.41; *SD* = 4.54)	79.60
Post-graduate	52 (*M* = 42.44; *SD* = 4.49)	15.60
Type of profession		
Student	251 (*M* = 28.38; *SD* = 7.81)	75.10
Employed	83 (*M* = 29.23; *SD* = 8.10)	24.90

*M is the average age of the participants. SD is the standard error for the age of the participants*.

### Procedure and Questionnaire

Our research was conducted using online questionnaires. This study used the contextual retrospective method to instruct the participants to recall and imagine that they were in a specific period of the COVID-19 pandemic. After situation priming in each period, they were then asked to answer the relevant questionnaires. All the questionnaires were self-compiled and calculated using a 7-point Likert rating tasks (Harpe, 2015; Joshi et al., 2015).

Our questionnaire consisted of three sub-questionnaires on the early, middle, and stable periods of the COVID-19 outbreak. Each sub-questionnaire was mainly composed of three parts. The first part evaluated an individual's mental state and the pandemic situation during this period. Mental state was measured using four items (*anxiety, pressure, fear, and tension*) with each item ranging from 1 (*not at all*) to 7 (*very much*). The average score of the four items was used as the mental state rating, and Cronbach's alpha for the four items was 0.95. Regarding the pandemic situation, we mainly evaluated it using two items (“*The current situation is uncontrollable”* and “*I am very confident in pandemic contro*l”) with each item ranging from 1 (*strongly disagree*) to 7 (*strongly agree*).

The second part mainly measured the characteristics of the publicity slogans of this period. The characteristics of publicity were mainly evaluated based on credibility, authority, funniness, and likability with each item ranging from 1 (not at all) to 7 (very much).

The third part mainly evaluated the feeling after reading the publicity slogans. The change in feelings after reading the slogan (*emotions, cognition, and behavior*) was scored on seven-point Likert rating tasks ranging from 1 (*strongly disagree*) to 7 (*strongly agree*). Among them, emotions mainly evaluated the change in participants' emotional arousal, which was mainly composed of three items. Cronbach's alpha of the three items was 0.91. Cognition mainly evaluated the change in participants' active acceptance and regulatory behavior, which was mainly composed of three items. Cronbach's alpha of the three items was 0.81. Behavior mainly evaluated whether the participant was likely to actively disseminate information to others. It was mainly composed of three items. Cronbach's alpha of the three items was 0.83. The Cronbach's alpha of the three measurement factors were all >0.8, so our self-compiled questionnaires were effective. Last, participants were asked to report their gender, age, educational background, occupation, and location.

## Results

### Evaluation of the Characteristics of the Two Forms of Publicity

Participants were asked to rate the two forms of publicity on authority, credibility, funniness and likability SPSS 23.0 software was used to conduct paired sample *T*-test for the evaluation results so as to distinguish between the two forms of publicity on the basis of the four characteristics. Specific results were shown in Table 3.

**Table 3:** Results of statistical differences in four characteristics between the two forms of publicity (general publicity and creative publicity).

**Characteristics**	**Paired Differences (General Publicity-Creative Publicity)**				
	**Mean**	**Std. Deviation**	**t**	**Sig**	**Cohen's d**
Authoritative	0.72	0.87	15.43	0.000	0.60
Credibility	0.52	0.71	13.78	0.000	0.49
Funniness	−1.11	1.16	−17.73	0.000	0.83
Likability	−0.22	0.91	−4.44	0.000	0.18

The statistical results showed that general publicity was evaluated to be more authoritative than creative publicity with *paired-t*_(333)_ = 15.43, *p* < 0.001, Cohen's *d* = 0.60. Moreover, the credibility of general publicity was higher than that of creative publicity, and the difference was significant [*paired-t*_(333)_ = 13.78, *p* < 0.001, Cohen's *d* = 0.49]. However, it was found that the creative publicity was funnier than general publicity, and the difference was significant [*paired t*_(333)_ = 17.73, *p* < 0.001, Cohen's *d* = 0.83]. Moreover, participants also liked creative publicity more than general publicity, and the difference was significant [paired-*t*_(333)_ = 4.44, *p* < 0.001, Cohen's *d* = 0.18].

### Evaluation of Mental States and Pandemic Situation

When evaluating mental states and the pandemic situation, a within-subjects design was adopted. One participant simultaneously evaluated the three different stages of the COVID-19 outbreak (early, middle, and stable period). Therefore, a single-factor three-level repeated-measures analysis of variance was performed on the results. The specific results were as follows:

#### Mental States

A repeated-measures analysis of variance (ANOVA) was conducted on the mental states of people during different periods of the COVID-19 outbreak, and significant differences in mental states were found during these periods [*F*_(2,332)_ = 412.38, *p* < 0.001, η_*p*_^2^ = 0.71]. The Bonferroni *post-hoc* test revealed that the mental states of people in the stable period of the COVID-19 outbreak (*M* = 2.99, *SD* = 1.58) were significantly better than those in the early (*M* = 4.97, *SD* = 1.45) and middle (*M* = 4.91, *SD* = 1.48) periods, but there was no significant difference between the early and middle periods of the COVID-19 outbreak.

#### Pandemic Situation

A repeated-measures ANOVA was performed for situational uncontrollability during different periods of the COVID-19 outbreak. Significant differences were found [*F*_(2,332)_ = 238.97, *p* < 0.001, η_*p*_^2^ = 0.58]. The Bonferroni *post-hoc* test revealed that the early (*M* = 4.49, *SD* = 1.68), middle (*M* = 4.27, *SD* = 1.72), and stable periods *(M* = 2.52, *SD* = 1.64) of the COVID-19 outbreak were significantly different, and they showed a significantly diminishing state—that is, people became increasingly optimistic about the situation of the pandemic throughout the development of COVID-19.

A repeated-measures ANOVA on epidemic control confidence showed that confidence in epidemic control was also significantly different during different periods of COVID-19 outbreak [*F*_(2,332)_ = 103.41, *p* < 0.001, η_*p*_^2^ = 0.38]. The Bonferroni *post-hoc* test revealed that the early (*M* = 4.74, SD = 1.60), middle (*M* = 4.94, *SD* = 1.56), and stable periods *(M* = 6.05, *SD* = 1.12) of the COVID-19 pandemic were significantly different. People were becoming increasingly confident in defeating the pandemic as it progressed.

### Effects of Two Forms of Publicity on Emotion, Cognition, and Behavior

To examine the influence of different publicity forms and different periods of COVID-19 on people's emotions, cognition and behavior, a within-subjects experimental design was adopted. We mainly investigated the effects of two independent variables (publicity forms, periods) on people's emotion, cognition, and behavior. All the results were analyzed by repeated measures ANOVA using SPSS23.0 software. The specific results were as follows:

#### Emotional Arousal

A 2 (forms of publicity: creative publicity, general publicity) × 3 (periods: early, middle, stable) repeated-measures ANOVA was conducted. The main effects of publicity [*F*_(1,333)_ = 21.81, *p* < 0.001, η_*p*_^2^ = 0.06] and periods [*F*_(2,332)_ = 23.08, *p* < 0.001, η_*p*_^2^ = 0.12) were both significant. More importantly, there was a significant interaction between the forms of publicity and the periods [*F*_(2,332)_ = 9.59, *p* < 0.001, η_*p*_^2^ = 0.05]. *Post-hoc* comparisons revealed that creative publicity more easily induced emotional arousal than general publicity, and this effect mainly appeared in the early [*F*_(1,333)_ = 16.30, *p* < 0.001, η_*p*_^2^ = 0.05] and middle periods [*F*_(1,333)_ = 29.04, *p* < 0.001, η_*p*_^2^ = 0.08] of the COVID-19 outbreak. The stable period of the COVID-19 outbreak failed to reveal a significant difference (*p* = 0.518).

#### Willingness to Regulate Behavior

A 2 (forms of publicity: creative publicity, general publicity) × 3 (periods: early, middle, stable) repeated-measures ANOVA was conducted for the willingness to regulate behavior. There was a significant main effect of period [*F*_(2,332)_ = 6.69, *p* = 0.001, η_*p*_^2^ = 0.04]. *Post-hoc* comparisons showed that, in the middle of the COVID-19 outbreak, people's willingness to regulate behavior was significantly higher than in the early and stable periods [*t*_(333)_ = 3.17, *p* = 0.002, *t*_(345)_ = 2.86, *p* = 0.005]. More importantly, a significant interaction between publicity and periods [*F*_(2,332)_ = 15.03, *p* < 0.001, η_*p*_^2^ = 0.08] was discovered. *Post-hoc* comparisons showed that, in the early period of the COVID-19 outbreak, general publicity made people more willing to regulate their behavior [*F*_(1,333)_ = 10.80, *p* = 0.001, η_*p*_^2^ = 0.03]. However, in the middle [*F*_(1,333)_ = 10.26, *p* = 0.001, η_*p*_^2^ = 0.03] and stable periods [*F*_(1,333)_ = 7.60, *p* = 0.006, η_*p*_^2^ = 0.02], creative publicity was more likely to play a role in regulating behavior.

#### Willingness to Actively Disseminate Information

A 2 (forms of publicity: creative publicity, general publicity) × 3 (periods: early, middle, stable) repeated-measures ANOVA was conducted for the willingness to actively disseminate information. There was a main effect of period [*F*_(2,332)_ = 24.49, *p* < 0.001, η_*p*_^2^ = 0.13]. *Post-hoc* comparisons found that, in the early period of the COVID-19 outbreak, willingness to actively disseminate information was significantly lower than in the middle and stable periods [*t*_(333)_ = −5.60, *p* < 0.001, *t*_(345)_ = −5.11, *p* = 0.001] while the difference between the middle and stable periods was not significant (*p* > 0.05). In addition, the interaction of publicity and periods was also significant [*F*_(2,332)_ = 5.77, *p* = 0.003, η_*p*_^2^ = 0.03]. *Post-hoc* comparisons showed that, in the early period of COVID-19, people were more willing to actively disseminate information using a general publicity form [*F*_(1,333)_ = 4.38, *p* = 0.04, η_*p*_^2^ = 0.01]. In contrast, in the middle period of the COVID-19 outbreak, creative publicity was more effective in inspiring people to disseminate information [*F*_(2,332)_ = 5.23, *p* = 0.023, η_*p*_^2^ = 0.02]. During the stable period of the COVID-19 outbreak, there was no difference between the two forms of publicity (*p* = 0.650).

**Figure 2:**
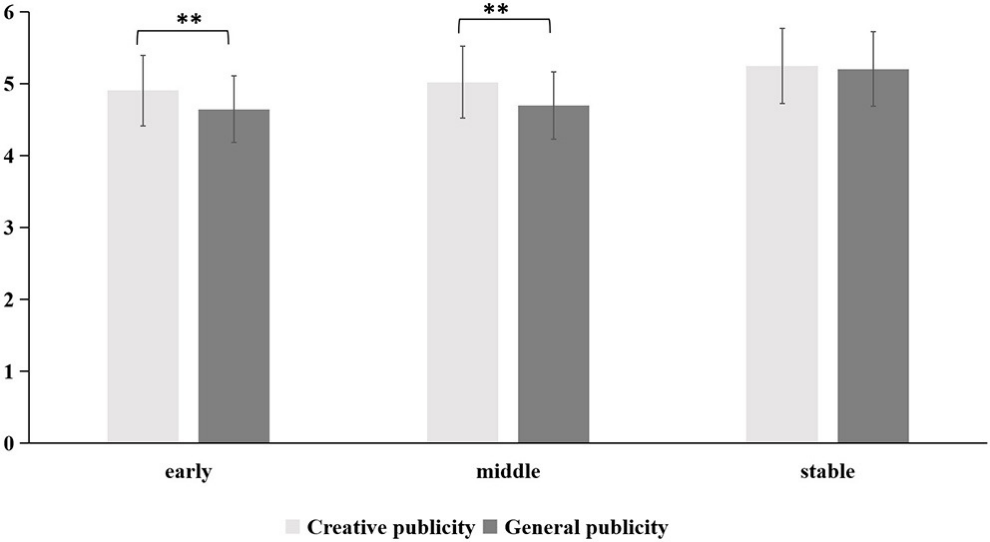
The influence of creative and general publicity on people's emotional arousal during the three periods of the COVID-19 outbreak (early, middle, and stable). Statistically significant differences are marked by *; *p < 0.05; **p < 0.01.

**Figure 3:**
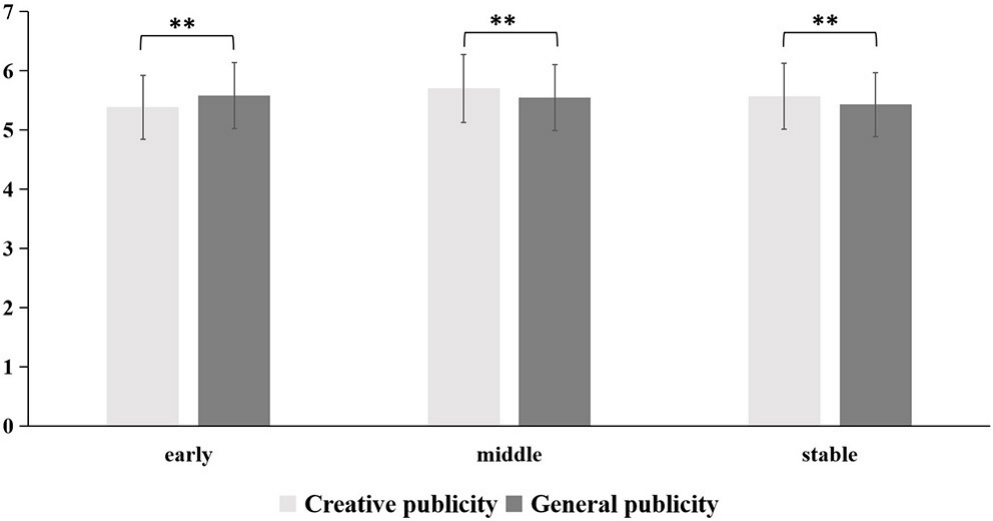
The influence of creative and general publicity on people's willingness to regulate behavior during the three periods of the COVID-19 outbreak (early, middle, and stable). **represents a significant difference at the level of 0.01, *P* ≤ 0.01.

**Figure 4:**
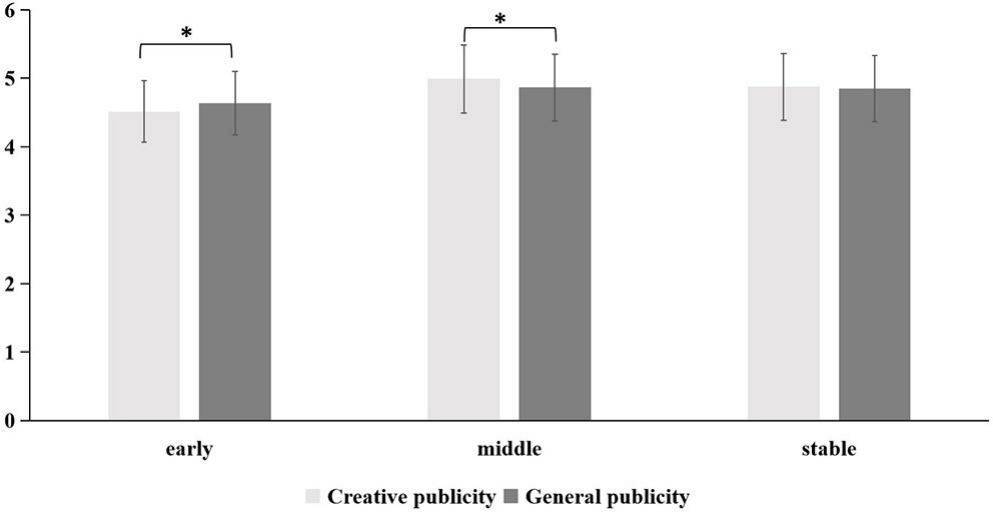
The influence of creative and general publicity on people's willingness to actively disseminate information during the three periods of the COVID-19 outbreak (early, middle, and stable). *represents significant difference at 0.05 level, *p* < 0.05.

In summary, our research found that creative and general publicity had differing effects on people's emotions, cognition, and behavior in different periods of the COVID-19 outbreak. The specific differences were shown in Figures 2–4.

## Discussion

The current study examines the role of creative text publicity based on poetry and general publicity during different periods of the COVID-19 outbreak in China. In the early period of the outbreak, the public's overall mental state was very poor and showed more negative emotions such as anxiety and fear. In particular, the “Wuhan lockdown” event led to people's overestimation of the uncontrollability of the pandemic situation and low confidence in its control. At this stage, creative publicity significantly affected people's emotional arousal and played a role in alleviating people's negative emotions. Creative publicity was more interesting because of its poetic language, so it was easier for people to have positive emotional experiences and improve their mental state. As shown in Figure 2, the effect of creative publicity on people's emotions lasted until the middle period of the COVID-19 outbreak. However, general publicity significantly affected people's willingness to regulate behavior and actively disseminate information about the epidemic. This result was unexpected and inconsistent with the research hypothesis in the present study. In previous studies, creative publicity had a more significant impact on people's cognition and behavior than general publicity; in the early period of the COVID-19 outbreak in our study, the opposite result appeared. This may be because general publicity was considered more authoritative and credible than creative publicity.

Helsloot and Ruitenberg (2004) proposed that the first stage of natural disasters is the early warning stage. At this stage, the most critical task is to encourage citizens to take the initiative to respond. After the occurrence of natural disasters, citizens will receive a large amount of disaster-related information, such as the number of casualties and protective measures, and they need to judge whether the information is true and credible and whether the proposed measures are effective and then act on whether to take measures (Perry, 1983). At this stage, citizens' responses to natural disasters were mainly determined by two factors: the credibility and authority of the information sources. In other words, the credibility of the publicity information and the authority of the source were more important than the form of publicity in the early period of COVID-19. On the other hand, the information in the early period of the COVID-19 outbreak was overwhelming, making it difficult for people to discern the authenticity of information. In this case, people were more likely to choose authoritative information to judge the current situation and make the most favorable choice for themselves. The information conveyed by the government was mostly in the form of documents, official records, and other general forms that typically indicate higher authority. Therefore, when the publicity theme was consistent, people were more likely to choose the content of general publicity with authority to guide and regulate their behaviors and actively disseminate the related information. This statement is also consistent with our finding that participants judged the authority and credibility of general publicity to be higher.

Our findings showed that, in the middle period of the COVID-19 outbreak, people's mental state was similar to that in the early period with anxiety and fear still dominating, but they had gained more understanding of the pandemic and had become more confident regarding controlling it. In addition, creative publicity was more likely to arouse emotions, prompt people to actively disseminate publicity information, and then regulate behaviors. This was consistent with the research hypothesis in the present study. Helsloot and Ruitenberg (2004) proposed that, in the acute stage of a crisis, the main mental state of citizens is panic and fear but that this stage is also a critical period for crisis management. In the middle of the COVID-19 outbreak in China, as the number of new cases increased daily, many cities began to implement the “city lockdown” policy to fight the spread of the pandemic. However, the Spring Festival was approaching at that time, and the Chinese have the custom of visiting relatives and friends during that festival, so they would have to give up their deep-rooted custom to support the “home quarantine” policy. In the face of sudden changes, getting people to understand, accept, and implement national epidemic prevention policies effectively became the key to success.

As one of the outstanding Chinese cultural traditions, Chinese poetry has a long history with its written form discovered as early as 1000 BC (Gao and Guo, 2018). Poetry usually originates from people's daily lives and has a good mass foundation and can be spread quickly through word of mouth due to its rhythm. Therefore, many local Chinese governments tried to incorporate poetry into publicity to motivate people to implement the national epidemic prevention policy. It turns out in our research that creative publicity based on poetic style was more popular, and this kind of method has achieved remarkable results. Compiling the publicity content into poems or writing propaganda slogans based on a poetic style can improve people's emotions, promote people's awareness of the pandemic, and inspire them to actively spread related information about COVID-19. On the contrary, in some other countries, while the advocacy of “wear a mask” and “home quarantine” was also implemented during the severe period of the pandemic, the effect was not obvious, which may have been related to the use of different methods of publicity. This inspired us to believe that it may be more effective to diffuse national epidemic prevention measures and knowledge to citizens through the creative publicity forms that people prefer and accept in the middle period of COVID-19.

During the stable period of the COVID-19 pandemic, creative publicity was more effective for people to regulate their behaviors. This effect lasted from the middle to the stable period of the outbreak, indicating that the cognitive change of creative publicity in people was sustained and significant. This was consistent with the results of research on creative advertising (Ahn et al., 2012; Reinartz and Saffert, 2013; Sameti and Khalili, 2017). Creative and general publicity both had better effects on emotional arousal and willingness to disseminate information, although the difference was not significant. This result was unexpected and inconsistent with the research hypothesis in the present study. This may be related to the social environment during the pandemic's stable period and people's perception of it. When the pandemic had reached a stable period, it had been brought under control to a certain extent but had not ended. Although people's mental states had improved, epidemic prevention and control had become a part of people's daily lives. People were always alert to the pandemic in terms of cognition. Therefore, no matter what kind of publicity form was adopted, people would be aroused by information about the pandemic and would actively disseminate any information to prevent the rebound of the pandemic. Helsloot and Ruitenberg (2004) proposed that this stage of a disaster is called the recovery stage. Therefore, during the stable period, people began to resume work and production, and China's economy began to recover. During this period, the government should intensify publicity efforts to keep people alert to the pandemic at all times.

In conclusion, based on the study of the COVID-19 pandemic, in different periods of natural disasters or crisis events, governments or medical departments should use a combination of creative and general publicity to impact people's emotions, cognition, and behavior. Creative publicity forms are novel and interesting, have a great impact on people's emotions and can relieve psychological pressure. Therefore, the best choice is to adopt creative publicity forms for self-protection, medical knowledge, and other contents, which can not only improve people's emotional state but also play a role in the transmission of knowledge. General publicity is more authoritative and credible and was mainly used in the early period of crisis. The emphasis and effect of the two forms of publicity differed. According to the emotional, cognitive, and behavioral needs of people during different events, the characteristics of the two forms of publicity were combined to determine the best effect.

## Limitations

As far as we know, this study is the first attempt to introduce creative publicity into crisis events and divide the COVID-19 pandemic in China into different periods, but some limitations still need to be mentioned. First, there are many other forms of creative publicity in addition to text publicity, such as images, video, and graphics. However, this study only selected creative text publicity based on the poetic style, which may, to some extent, limit the scope of the conclusion. In future studies, a variety of creative publicity forms should be explored to better serve the public in natural disasters such as the COVID-19 pandemic. Second, our study mainly examines the different periods of the COVID-19 outbreak in China. However, the classification of periods of the COVID-19 pandemic may differ in different countries. Therefore, if more countries can be included in future studies, the conclusions obtained will be more convincing. Thirdly, this study was only conducted in China, and its results may be limited by geography. In the future, a comparative study between different countries may yield more generalizable results.

## Conclusion

The study revealed that creative publicity had differing effects in different periods of the COVID-19 outbreak in China. Specifically, in the early period of the outbreak, creative publicity had a significant impact on individuals' emotional arousal; general publicity effectively regulated individuals' behavior and actively disseminated information about the pandemic to people around them. Creative publicity in the middle period of the outbreak significantly affected individuals' emotional arousal, regulated their behaviors, and improved willingness to actively disseminate information. In the stable period, creative publicity was superior to general publicity in regulating individuals' behavior. Creative publicity and general publicity had different effects in different periods of the COVID-19 outbreak, which may relate to the characteristics of the publicity form and the psychological condition of people in different periods.

Therefore, in the early period of crisis, the authority of publicity can most affect people's cognition and behavior. At this time, the government and other official departments ought to strengthen communication with the public and issue various crisis-related documents to achieve the purpose of rapid communication with people. At the same time, the results of the study also illustrated the role of information openness in crisis events. People's reactions were most influenced by information from authorities. In the middle of the crisis, which was also the critical period of the crisis, creative publicity can help effortlessly achieve the purpose of efficient communication with people. Moreover, it can quickly organize the public to deal with the crisis, and reduce the necessary losses. At this time, creative publicity can have a comprehensive impact on people's emotion, cognition, and behavior. With more publicity, people can get through the crisis more efficiently.

To sum up, choosing a more efficient medium of communication depending on the period of the crisis events can assist in dealing with the crisis. Creative publicity provides us with a good communication strategy and method.

## Data Availability Statement

The raw data supporting the conclusions of this article will be made available by the authors, without undue reservation.

## Ethics Statement

All experimental procedures were reviewed and ethically approved by the Ethics Council of Central China Normal University. The patients/participants provided their written informed consent to participate in this study.

## Author Contributions

DJ: conceptualization, methodology, formal analysis, investigation, wrote-original draft, wrote—review, and editing. CS: investigation, wrote—review, and editing. ZZ: conceptualization, methodology, supervision, review, and editing. QZ: investigation and methodology. QY: supervision, review, and editing. GL: methodology. YW: investigation. All authors contributed to the article and approved the submitted version.

## Conflict of Interest

The authors declare that the research was conducted in the absence of any commercial or financial relationships that could be construed as a potential conflict of interest.
